# Editorial: Computer vision mechanisms for resource-constrained robotics applications

**DOI:** 10.3389/frobt.2025.1680098

**Published:** 2025-09-02

**Authors:** Rui Pimentel de Figueiredo, Christian Limberg, Lorenzo Jamone, Alexandre Bernardino

**Affiliations:** 1 Department of Mechanical and Production Engineering, Aarhus University, Aarhus, Denmark; 2 National Institute of Informatics (NII), Chiyoda-ku, Tokyo, Japan; 3 Department of Computer Science, University College London (UCL), London, United Kingdom; 4 Institute For Systems and Robotics, Instituto Superior Tecnico, University of Lisbon, Lisbon, Portugal

**Keywords:** perception, computer vision, robotics, resource-constrained, artificial intelligence

## 1 Key contributions in the special issue

In the evolving landscape of robotics, the challenge of deploying intelligent systems in real-world environments under limited computational and energy budgets has become increasingly important. The Research Topic of *Frontiers in Robotics and AI*—*“Computer Vision Mechanisms for Resource-Constrained Robotics Applications”*, gathers a series of innovative contributions that directly address this challenge. The Research Topic not only reflects the state of the art in efficient robotic perception, but also channels bio-inspired models, minimal computing principles, and hardware-constrained innovation to define a compelling path forward.

The premise of the Research Topic is grounded in a vital observation: although computer vision has reached extraordinary levels of accuracy and generalization through deep learning and high-performance sensors, these solutions are often tethered to expensive and power-hungry hardware and computationally complex algorithms. Autonomous robots intended for field deployment—whether aerial drones, underwater vehicles, or mobile ground units—frequently face bandwidth, energy, and compute bottlenecks. Thus, vision mechanisms designed with constraint-awareness are not merely desirable, but necessary.

One prominent contribution to the Research Topic is Singh et al.’s framework on *minimal perception*, which seeks to enable robotic autonomy by reducing perceptual complexity to its essential components Singh et al. Drawing inspiration from ecological psychology and AI, the authors design systems where perception is not treated as a comprehensive reconstruction of the world, but rather as a dynamic filter tuned to task relevance. The approach is validated through simulation and robot experiments, demonstrating that sparse and compressed sensory signals can support goal-directed behaviors such as obstacle avoidance and navigation with minimal computational overhead.

Another standout is Van Opstal et al.’s biomimetic robotic eye, which introduces a six-degree-of-freedom platform capable of replicating human-like saccadic movements. The design merges active vision control with neurophysiological plausibility, offering both an experimental platform for neuroscientific hypotheses and a roadmap for embodied perception in robotics Van Opstal et al. The project brings to life ideas proposed in earlier models of oculomotor control, notably by Robinson and Sparks, but with real-time hardware precision and active visual fixation.

Kalou et al. contribute with their work on head-centric representation of 3D shapes, reformulating shape perception as a process guided by an embodied fixation system Kalou et al. Their compact encoding scheme capitalizes on spatial priors gained from head-centered visual exploration, echoing Gibson’s ecological theory of vision Gibson (1979) and early active vision paradigms.

On the sensor innovation front, Ribeiro-Gomes et al. advocate for event-based cameras as foundational tools in embedded robotics. Their integration of asynchronous feature tracking into a visual-inertial odometry (VIO) framework demonstrates improved latency and energy efficiency compared to conventional methods Ribeiro-Gomes et al. These contributions collectively reflect a growing interest in both algorithmic minimalism and bio-inspired adaptation.

## 2 Future directions and editorial perspective

The research compiled in this Research Topic reflects a broader shift in the field: away from monolithic perception pipelines and toward adaptive, embodied, and task-sensitive vision systems. A key theme is the resurgence of the perception-action loop, long emphasized by ecological psychology and active vision pioneers such as Aloimonos et al. (1988) and Gibson (1979), but now reinvigorated through hardware-aware and task-constrained implementations.

First, there is a growing recognition of the need for perception systems that dynamically adapt their computational load. Runtime-adaptive pipelines—capable of modulating neural network size, activation depth, or sensor resolution in response to context—could become essential for sustained autonomy in resource-constrained environments.

Second, spiking neural models and neuromorphic circuits represent a biologically plausible and energy-efficient alternative to dense convolutional networks. This trend aligns with broader developments in transprecision computing and event-driven processing Delbruck and Lichtsteiner (2007); Sze et al. (2017), offering pathways for ultra-low-power robotics.

A third Frontier lies in the development of learning-based attention strategies. While classical attention mechanisms in vision prioritize computational saliency, emerging work explores end-to-end learning of attention policies that guide both perception and action. Reinforcement learning, meta-learning, and unsupervised learning strategies may enable robots to allocate their limited perceptual resources more effectively—particularly when paired with real-time hardware feedback on power and latency.

Finally, the field would benefit greatly from standardized benchmarks designed specifically for resource-constrained robotics. While robust datasets exist for general tasks like SLAM or autonomous driving, few evaluate performance under explicit constraints such as degraded sensing, memory limits, or energy caps. Establishing such benchmarks could spur more targeted innovation and help consolidate this rapidly growing subfield.

In terms of real-world impact, the relevance of resource-constrained perception extends well beyond research labs. In industrial robotics, especially in domains such as manufacturing, logistics, and infrastructure inspection, there is a growing demand for agile and power-efficient robots that can operate reliably in bandwidth-limited and dynamic environments. Many of these applications involve mobile platforms or wearable systems where energy and compute budgets are tightly constrained. Vision systems that adaptively throttle processing, or that rely on asynchronous and event-based sensing, are particularly well-suited for tasks such as real-time anomaly detection, autonomous warehouse navigation, and human-robot collaboration on production lines.

Moreover, the advances discussed in this Research Topic intersect meaningfully with the development of bio-inspired and humanoid robots. Platforms modeled after human morphology benefit immensely from perception systems that mimic the selective, task-dependent nature of biological vision. For example, the implementation of saccadic control, head-centric representation, and embodied attention strategies aligns naturally with the perceptual architectures found in humanoid robots. Such robots—whether designed for eldercare, physical rehabilitation, or disaster response—require lightweight and efficient perception pipelines that can operate within strict power, latency, and mobility constraints dictated by their human-centric form and mission requirements.

## 3 Conclusion

From biologically inspired eye movements to minimal perception frameworks and event-based feature tracking, the contributions point toward a future where efficiency and embodiment are not peripheral concerns, but rather central pillars of robotic intelligence, particularly as robotics moves further into industrial, assistive, and human-interactive domains.

This Research Topic offers a compelling survey of the innovations shaping vision for resource-constrained robotics. From biologically inspired eye movements to minimal perception frameworks and event-based feature tracking, the contributions point toward a future where efficiency and embodiment are not peripheral concerns, but rather central pillars of robotic intelligence.
